# Synthesis of magnolol and honokiol derivatives and their effect against hepatocarcinoma cells

**DOI:** 10.1371/journal.pone.0192178

**Published:** 2018-02-07

**Authors:** Margherita Maioli, Valentina Basoli, Paola Carta, Davide Fabbri, Maria Antonietta Dettori, Sara Cruciani, Pier Andrea Serra, Giovanna Delogu

**Affiliations:** 1 Department of Biomedical Sciences, University of Sassari, Sassari, Italy; 2 Laboratory of Molecular Biology and Stem Cell Engineering, National Institute of Biostructures and Biosystems, Bologna, Italy; 3 Institute of Neurogenetics and Neuropharmacology, National Research Council, Monserrato, Cagliari, Italy; 4 Centre for Developmental Biology and Reprogramming (CEDEBIOR), Department of Biomedical Sciences, University of Sassari, Sassari, Italy; 5 Department of Biotechnology, University of Natural Resources and Life Sciences Vienna, Vienna, Austria; 6 Institute of Biomolecular Chemistry, National Research Council, Sassari, Italy; 7 Department of Clinical and Experimental Medicine, University of Sassari, Sassari, Italy; National Research Council of Italy, ITALY

## Abstract

The hepatocellular carcinoma is one of the most common malignant tumour with high level of mortality rate due to its rapid progression and high resistance to conventional chemotherapies. Thus, the search for novel therapeutic leads is of global interest. Herein, a small set of derivatives of magnolol **1** and honokiol **2**, the main components of *Magnolia grandiflora* and *Magnolia obovata*, were evaluated in *in vitro* assay using tumoral hepatocytes. The pro-drug approach was applied as versatile strategy to the improve bioactivity of the compounds by careful transformation of the hydroxyl groups of magnolol **1** and honokiol **2** in suitable ester derivatives. Compounds **10** and **11** resulted to be more potent than the parental honokiol **2** at concentration down to 1 μM with complete viability of treated fibroblast cells up to concentrations of 80 μM. The combination of a butyrate ester and a bare phenol-OH group in the honokiol structure seemed to play a significant role in the antiproliferative activity identifying an interesting pharmacological clue against hepatocellular carcinoma.

## Introduction

Hepatocellular carcinoma (HCC) is the second leading cause of cancer death worldwide with an estimated incidence of half a million new cases per year around the world [1, 2]. HCC is a frequent complication of liver cirrhosis. Major risk factors for the development of HCC are obesity, diabetes mellitus, non-alcoholic fatty liver disease, excessive alcohol consumption and dietary aflatoxin B1 [3]. Nowadays, there is no definitive curative treatment for HCC; moreover, treatment and management modalities exist with differing advantages and disadvantages [4]. Hence, novel therapeutics are urgently needed for the treatment of HCC patients.

Naturally-occurring polyphenols are a wide class of plant secondary metabolites, which promote reproduction and protect the plant against hexogen agents and radiations [5, 6]. They are well known as a dietary supplement [7] or cosmetic additives [8]. In addition, they are used as starting material for the preparation of new therapeutic agents against many type of diseases [9]. Polyphenols with a flexible structure have attracted considerable attention as effective ligands for receptor molecules involved in the aetiology of many diseases acting by modulating binding affinity in a wide variety of proteins [10, 11]. Among them, hydroxylated biphenyls have an important role due to their unique pharmacophore structure formed of two aromatic rings bridged by a single C-C bond [12, 13]. This structural feature allows for the activation of a sufficient number of interactions with a variety of sites present on proteins surfaces [14]. In the presence of a steric hindrance by substituents close to the single C-C bond, a selective chiral recognition occurs between the protein and the biphenyl structure. The axial chirality of biphenyls and in general, of biaryls, is a consequence of a hindered rotation around the aryl-aryl single bond, named atropisomerism [12]. As result, chiral recognition biphenyl-protein can influence the biological activity of the living organism. Although less effective, a chiral recognition might also occurs between protein and configurationally unstable biphenyls, due to the structural feature of the biphenyl pharmacophore. It is generally recognised that hydroxylated biphenyls are privileged structures able to contrast oxidative stress, a natural phenomenon of the cellular metabolism, often better than their corresponding parental mono-phenols [15, 16]. Antioxidant activity improves when the phenol-OH groups are located in *ortho* position to the single C-C bond bridging the two aromatic rings. Their activity is due to the easy abstraction of the first hydrogen of the phenol-OH group by a peroxyl radical and the formation of a stable intramolecular hydrogen bond between the second phenol-OH group and phenoxyl radical [17]. The presence of proper substituents in *ortho* position to the phenol-OH group can influence the intramolecular hydrogen bonding and, thus, the stabilisation of the generated phenoxyl radical [18]. Considering the importance of the biphenyl pharmacophore, the synthesis of hydroxylated biphenyls, whose structure resembles that of the natural compound, represents a novel target of research in the field of new therapeutic agents [19–22].

Magnolol (**1**, 5,5’-diallyl-2,2’-dihydroxybiphenyl) and honokiol (**2**, 5,5’-diallyl-2,4’-dihydroxybiphenyl) are hydroxylated biphenyls isolated from the root and stem bark of *Magnolia officinalis*, a plant widely distributed in China, Japan, and South Korea [23]. Magnolol **1** and honokiol **2** have shown muscle relaxant, neuroprotective, anti-oxidative, anti-atherosclerosis, anti-inflammatory and anti-microbial effects [24–26]. Recent studies have shown that magnolol **1** and honokiol **2** exhibits anti-cancer properties by inhibiting proliferation, inducing differentiation and apoptosis, suppressing angiogenesis, countering metastasis, and reversing multi-drug resistance [27–33]. In the search for therapeutic approaches against hepatocellular carcinoma, non-alcoholic fatty liver disease, hepatic fibrosis and xenobiotics-induced liver injury, recent studies have investigated the use of both magnolol **1** and, especially, honokiol **2** [34]. They inhibit STAT3 signal and dysregulate KRT6 gene expression, both of which are hepatocytes key factors and modulate the NF-kB pathway, which protects cells from a variety of oxidative stresses [35–38]. However, the poor aqueous solubility of magnolol **1** and honokiol **2**, and in general, of naturally-occurring polyphenols, has hampered the clinical applications of this kind of compounds, which possess a wide variety of biological activities. In order to overcome this issue, encapsulation of honokiol have been assessed [39, 40] and synthetic derivatives have been prepared [41].

The aim of the present work was to develop derivatives of magnolol **1** and honokiol **2** more water-soluble and highly potent in low doses compared to the parental compounds, and against tumoural hepatocytes proliferation. The pro-drug approach [42] was applied as versatile strategy to improve the bioactivity of the compounds by transformation of the hydroxyl groups of magnolol **1** and honokiol **2** in an ester functional group with an acetate and a butyrate, respectively. Subsequently, the ester group would undergone *in vivo* biotransformation through chemical or enzymatic cleavage, thus favouring the delivery of the active compound with a higher yield. Increase effectiveness was expected with acetate derivatives of magnolol **1** and honokiol **2** as compared to the parental compounds. In the case of the butyrate derivative, a mutual pro-drug would be formed by the presence of butyric acid, a known inducer of fibroblast growth factor 21 in liver [43, 44]. We have previously shown that butyrate, in a preparation with a glycoconjugate ester, was able to influence cells fate eliciting cardiogenic commitment of both murine embryonal stem cells, and human mesenchymal stem cells from different sources, also affecting myocardial regeneration in infarcted rat hearts [45–47].

Based on these results and in continuation of our studies on the synthesis of bioactive hydroxylated biphenyls from natural-occurring compounds [19, 20, 48, 49], we aimed at investigating a major effect of acetate and butyrate derivatives of magnolol **1** and honokiol **2**, respectively, supposing a mutual interaction able to affect cellular processes, such as cell cycle control and cell proliferation, specifically in tumoural cells. Our compounds were tested on Hep2G cells, and hepatic cell line, normally used as hepatocarcinoma model, and on HFF1 cells, a fibroblast cell line, in order to identify the effects of different modifications of magnolol **1** and honokiol **2** specific only for tumoural cells, in order to exclude a general possible cytotoxic effect.

## Materials and methods

### General

Melting points are uncorrected. All ^1^H NMR and ^13^C NMR spectra were recorded on spectrometer Varian Mercury Plus operating at 399.93 MHz and 100.57 MHz, respectively. Chemical shifts are given in ppm (δ) and coupling constants in Hertz; multiplicities are indicated by s (singlet), d (doublet), t (triplet), ddt (doublet doublet triplet), m (multiplet) or series of m (series of multiplet). CDCl_3_, acetone-*d6*, were used as solvents as indicated below. Shifts are given in ppm relative to the remaining protons of the deuterated solvents used as internal standard (^1^H, ^13^C). Elemental analyses were performed using an elemental analyser Perkin-Elmer model 240 C. Ethanol (EtOH) grade 96% was used, dry acetone was freshly distilled from CaCl_2_ under N_2_. All reagents were of commercial quality and used as purchased from various producers (Sigma-Aldrich, Merck). Magnolol **1** and honokiol **2** were purchased from Chemos GmbH, Germany. Flash chromatography was carried out with silica gel 60 (230–400 mesh, Kiesgel, EM Reagents) eluting with appropriate solution in the stated v:v proportions. Analytical thin-layer chromatography (TLC) was performed with 0.25 mm thick silica gel plates (Polygram^®^ Sil G/UV_254_, Macherey-Nagel). The purity of all new compounds was judged to be >98% by ^1^H-NMR spectral determination. Solvents were used without additional purification or drying, unless otherwise noted.

### Synthesis of compounds

#### Compound 3

To a solution of magnolol **1** (2 g, 7.5 mmol) in dry acetone (20 mL) potassium carbonate (2.06 g, 15 mmol) was added under N_2_. The reaction mixture was stirred at rt for 10 min acetic anhydride (1.56 mL g, 16.5 mmol) was added and, after stirring at rt for 1 h, the solution was filtered and rotoevaporated to obtain a viscous oil that was purified by flash chromatography using dichloromethane as eluent. **Compound 3** [50]: (oil) (2.02 g, 85%): ^1^H NMR (CDCl_3_) δ 1.97 (s, 3H), 3.33 (d, *J* = 6.4 Hz, 4H), 5.09 (m, 4H), 5.80 (m, 2H), 6.94 (d, *J* = 8.4 Hz, 2H), 7.05 (d, *J* = 2.0 Hz, 2H), 7.5 (dd, *J* = 2.0, 8.4 Hz, 2H); ^13^C NMR (CDCl_3_) δ 20.76, 39.52, 116.22, 122.41, 128.84, 130.26, 131.24, 136.95, 137.70, 146.32, 169.48; C_22_H_22_O_4_: C, 75.41; H, 6.33; Found: C, 75.47; H, 6.35.

#### Compound 4

To a solution of magnolol **1** (2 g, 7.5 mmol) in dry acetone (20 mL) potassium carbonate (1.03 g, 7.5 mmol) was added under N_2_. The reaction mixture was stirred at rt for 10 min, acetic anhydride (0.71 mL g, 7.5 mmol) was added and, after stirring at rt for 1 h, the solution was filtered and rotoevaporated to obtain an viscous oil that was purified by flash chromatography using dichloromethane as eluent. **Compound 4** [50]: (oil) (2.11 g 90%): ^1^H NMR (CDCl_3_) δ 2.04 (s, 3H), 3.33 (d, *J* = 7.2 Hz, 2H), 3.43 (d, *J* = 7.2 Hz, 2H), 5.04–5.17 (series of m, 4H), 5.99 (m, 2H), 6.90 (d, *J* = 8.4 Hz, 1H), 6.95 (d, *J* = 2.4 Hz, 1H), 7.08 (dd, *J* = 2.4, 8.0 Hz, 1H), 7.15 (d, *J* = 8.4 Hz, 1H), 7.21 (d, *J* = 2.4 Hz, 1H), 7.26 (dd, *J* = 2.4, 8.0 Hz, 1H); ^13^C NMR (CDCl_3_) δ 20.62, 39.30, 39.56, 115.56, 116.28, 116.43, 122.90, 123.90, 129.56, 129.69, 130.09, 130.61, 131.83, 131.92, 136.75, 137.77, 138.70, 146.84, 151.28, 169.89; Anal. Calcd for C_20_H_20_O_3_: C, 77.90; H, 6.54; Found: C, 78.03; H, 6.56.

#### Compound 5

To a solution of honokiol **2** (2 g, 7.5 mmol) in dry acetone (20 mL) potassium carbonate (2.06 g, 15 mmol) was added under N_2_. The reaction mixture was stirred at rt for 10 min acetic anhydride (1.68 mL g, 16.5 mmol) was added and, after stirring at rt for 1 h, the solution was filtered and rotoevaporated to obtain a viscous oil that was purified by flash chromatography using dichloromethane: petroleum ether 1: 1 solution as eluent. **Compound 5** [22]: (oil) (1.94 g 74%) and a 75: 25 mixture of **Compound 6** + **Compound 7** (oil) (0.35 g, 15%).

**Compound 5**: ^1^H NMR (CDCl_3_) δ 2.09 (s, 3H), 2.32 (s, 3H), 3.35 (d, *J* = 6.8 Hz, 2H), 3.43 (d, *J* = 6.8 Hz, 2H), 5.07–5.15 (series of m, 4H), 5.96 (m, 2H), 7.05 (d, *J* = 8 Hz, 1H), 7.10 (d, *J* = 8.0 Hz, 1H), 7.20 (dd, *J* = 2.4, 8.0 Hz, 1H), 7.22 (d, *J* = 2.4 Hz, 1H), 7.29 (dd, *J* = 2.4, 8.0 Hz, 1H), 7.30 (d, *J* = 2.4, Hz, 1H); ^13^C NMR (CDCl_3_) δ 20.90, 20.95, 34.72, 39.63, 116.32, 116.42, 122.33, 122.79, 127.92, 128.70, 130.87, 130.93, 131.69, 133.79, 135.56, 135.75, 136.93, 138.24, 145.99, 148.35, 169.37, 169,62; Anal. Calcd for C_22_H_22_O_4_: C, 75.41; H, 6.33; Found: C, 75.46; H, 6.32.

#### Compounds 5, 6, 7

To a solution of honokiol **2** (1 g, 3.76 mmol) in dry acetone (20 mL) potassium carbonate (0.57 g, 3.76 mmol) was added at 0 °C under N_2_. The reaction mixture was stirred at RT for 30 min acetic anhydride (0.42 mL g, 4.13 mmol) was added and, after stirring at rt for 1 h, the solution was filtered and rotoevaporated to obtain an viscous oil that was purified by flash chromatography using petroleum ether:tetrahydrofurane 10:2 solution as eluent to obtain:, **compound 5** (oil) (0.22 g, 16%), **compound 6** (oil) (0.43 g, 36%), **compound 7** (oil) (0.14 g, 12%) and starting material (honokiol **2**) (0.16 g, 16%).

**Compound 6**: ^1^H NMR (CDCl_3_) δ 2.34 (s, 3H) 3.35 (m, 4H), 5.06–5.14 (series of m, 4H), 5.94 (m, 2H), 6.89 (d, *J* = 8.4 Hz, 1H), 6.99-7-09 (series of m, 2H), 7.14 (d, *J* = 8.4 Hz, 1H), 7.31-7-39 (series of m, 2H); ^13^C NMR (CDCl_3_) δ 20.95, 34.79, 39.38, 115.66, 115.99, 116.63, 123.04, 127.33, 128.27, 129.20, 130.34, 131.20, 132.25, 132.72, 135.44, 135.55, 137.73, 148.46, 150.90, 169.67; Anal. Calcd for C_20_H_20_O_3_: C, 77.90; H, 6.54; Found: C, 78.00; H, 6.52.

**Compound 7**: ^1^H NMR (CDCl_3_) δ 2.09 (s, 3H), 3.42 (m, 4H), 5.07–5.17 (series of m, 4H), 5.67 (bs, 1H), 5.97 (m, 2H), 6.79 (d, *J* = 8.0 Hz, 1H), 7.16 (d, *J* = 8.0 Hz, 1H), 7.15-7-20 (series of m, 4H); ^13^C NMR (CDCl_3_) δ 20.94, 34.89, 39.67, 115.59, 116.18, 116.34, 122.62, 125.40, 128.10, 128.15, 130.03, 130.77, 130.84, 134.30, 136.38, 137.06, 138.18, 145.98, 153.66, 169.90; Anal. Calcd for C_20_H_20_O_3_: C, 77.90; H, 6.54; Found: C, 78.02; H, 6.55.

#### Compounds 5–7: Microwave procedure

In a 30 mL glass pressure microwave tube, equipped with a magnetic stirrer bar, a solution of honokiol **2** (0.10 g, 0.38 mmol) in the presence of an inorganic base (1 equiv per -OH) and acetic anhydride (3 mL) was subjected to microwave irradiation as described in Table 1. Dichloromethane and water work-up of the reaction mixture lead to two phases that were separated. The organic phase, extracted with dichloromethane (2 x 30 mL) was dried over Na_2_SO_4_ and evaporated to afford a crude brown product that was analysed by ^1^H NMR.

**Table 1:** Microwave preparation of biphenyls 5–7.

Entry	base	6 : 7	(6 + 7) : 5
**1** ^a^	K_2_CO_3_	8 : 2	6 : 4
**2** ^b^	K_2_CO_3_	6 : 4	7 : 3
**3** ^a^	Cs_2_CO_3_	8 : 2	8 : 2
**4** ^c^	CaCO_3_	7 : 3	9 : 1

^a^: neat, T 55 °C, t 5ʹ, PW 60W.

^b^: in acetone, T 55 °C, t 10ʹ.

^c^: neat, T 100 °C, t 5ʹ, PW 60 W.

#### Compound 8

To a solution of magnolol **1** (1.0 g, 3.76 mmol) in dry acetone (30 mL) potassium carbonate (1.04 g, 7.52 mmol) was added under N_2_. The reaction mixture was stirred under reflux for 30 min. Butyryl chloride (0.80 g, 7.52 mmol) was added and, after stirring under reflux for 24 h, the solution was filtered and rotoevaporated to obtain a viscous oil that was purified by flash chromatography using dichloromethane as eluent. **Compound 8** (oil) (1.40 g 92%) and **Compound 9** (oil) (0.10 g 8%).

**Compound 8**: ^1^H NMR (CDCl_3_) δ 0.80 (t, *J* = 7.2 Hz, 6H), 1.51 (m, 4H), 2.53 (t, *J* = 7.2 Hz, 4H), 3.38 (d, *J* = 6.8 Hz, 4H), 5.07 (m, 4H), 5.96 (m, 2H), 7.04 (d, *J* = 8.0 Hz, 2H), 7.1 (d, *J* = 2.0 Hz, 2H), 7.18 (dd, *J* = 2.0, 8.0 Hz, 2H); ^13^C NMR (CDCl_3_) δ 13.41, 18.15, 35.85, 39.45, 116.14, 122.34, 128.87, 130.41, 131.19, 137.01, 137.48, 146.44, 172.04; Anal. Calcd for C_26_H_30_O_4_: C, 76.82; H, 7.44; Found: C, 76.89; H, 7.38.

#### Compound 9

To a solution of magnolol (1.0 g, 3.76 mmol) in dry acetone (30 mL) potassium carbonate (0.52 g, 3.75 mmol) was added under N_2_. The reaction mixture was stirred under reflux for 30 min. Butyryl chloride (0.40 g, 3.75 mmol) was added and, after stirring under reflux for 24 h, the solution was filtered and rotoevaporated to obtain an viscous oil that was purified by flash chromatography using dichloromethane as eluent. **Compound 9** (oil) (0.87 g 79%), **Compound 8** (oil) (0.23 g 15%) and starting material (magnolol **1**) (0.06 g 6%). **Compound 9**: ^1^H NMR (CDCl_3_) δ 0.77 (t, *J* = 7.2 Hz, 3H), 1.47 (m, 2H), 2.27 (t, *J* = 7.2 Hz, 2H), 3.31 (d, *J* = 6.8 Hz, 2H), 3.42 (d, *J* = 6.8 Hz, 2H), 5.02–5.15 (series of m, 4H), 5.96 (m, 2H), 6.90 (d, *J* = 8.4 Hz, 1H), 6.92 (d, *J* = 2.0 Hz, 1H), 7.07 (dd, *J* = 2.0, 8.4 Hz, 1H), 7.09 (d, *J* = 8.4 Hz, 1H), 7.18 (d, *J* = 2.0 Hz, 1H), 7.25 (dd, *J* = 2.0, 8.4 Hz, 1H); ^13^C NMR (CDCl_3_) δ 13.34, 18.16, 35.86, 39.30, 39.55, 115.52, 116.29, 116.43, 122.84, 123.98, 129.67, 129.64, 130.07, 130.57, 131.83, 131.91, 136.73, 137.71, 138.66, 146.92, 151.27, 172.48; Anal. Calcd for C_22_H_24_O_3_: C, 78.54; H, 7.19; Found: C, 78.58; H, 7.18.

#### Compound 10

To a solution of honokiol **2** (1.0 g, 3.76 mmol) in dry acetone (30 mL) potassium carbonate (1.24 g, 9.02 mmol) was added under N_2_. The reaction mixture was stirred under reflux for 30 min. Butyryl chloride (0.96 g, 9.02 mmol) was added and, after stirring under reflux for 24 h, the solution was filtered and rotoevaporated to obtain an viscous oil that was purified by flash chromatography using petroleum ether: tetrahydrofurane 6: 1 mixture as eluent to obtain compound **10** (oil, 0.69 g 45%), compound **11** (oil, 0.09 g, 7%), compound **12** (oil, 0.35 g 28%) and starting material (honokiol **2**) (0.13 g, 13%).

**Compound 10** (oil): ^1^H NMR (CDCl_3_) δ 0.88 (t, *J* = 7.2 Hz, 3H); 1.08 (t, *J* = 7.2 Hz, 3H); 1.61 (m, 2 H); 1.84 (m, 2H); 2.35 (t, *J* = 7.2 Hz, 3H); 2.58 (t, *J* = 7.2 Hz, 3H); 3.34 (d, *J* = 6.8 Hz, 2H); 3.43 (d, *J* = 6.8 Hz, 2H); 5.12 (m, 4H); 5.96 (m, 2H); 7.05 (d, *J* = 8.0 Hz, 1H); 7.10 (d, *J* = 8.0 Hz, 1H); 7.21 (dd, *J* = 2.0 and 8.4 Hz, 1H); 7.23 (d, *J* = 2.0 Hz, 1H); 7.31 (m, 2H). ^13^C NMR (CDCl_3_) δ 13.51, 13.77, 18.24, 18.50, 34.70, 36.07, 36.16, 39.64, 116.26, 116.38, 122.29, 122.83, 128.04, 128.65, 130.93, 130.96, 131.60, 134.03, 135.52, 135.77, 137.01, 138.03, 146.10, 146.42, 171.88, 172.08. Anal. Calcd for C_26_H_30_O_4_ C, 76.82; H, 7.44; Found: C, 76.86; H, 7.42.

#### Compounds 10–12

To a solution of honokiol (1 g, 3.76 mmol) in dry acetone (30 mL) potassium carbonate (0.62 g, 4.51 mmol) was added under N_2_. The reaction mixture was stirred under reflux for 30 min. Butyryl chloride (0.48 g, 4.51 mmol) was added and, after stirring under reflux for 12 h, the solution was filtered and acetone evaporated to obtain a viscous oil that was purified by flash chromatography using petroleum ether: tetrahydrofurane 6:1 mixture as eluent to obtain **compound 10** (oil, 0.3 g 20%), **compound 11** (oil, 0.16 g, 13%), **compound 12** (oil, 0.36 g 28%) and starting material (honokiol) (0.32 g, 32%).

**Compound 11**: ^1^H NMR (acetone d_6_) δ 0.85 (t, *J* = 7.2 Hz, 3H); 1.57 (m, 2H); 2.36 (t, *J* = 7.2 Hz, 2H); 3.41 (m, 4H); 4.99–5.15 (series of m, 4H); 6.01 m, 2H); 6.90 (d, *J* = 8.4 Hz, 1H); 7.03 (d, *J* = 8.4 Hz, 1H); 7.11 (dd, *J* = 2.4 and 8.0 Hz, 1H); 7.17 (m, 2H); 7.21 (d, *J* = 2.4 Hz, 1H); 8.04 (s, OH). ^13^C NMR (acetone d6) δ 12.83, 17.90, 34.05, 35.55, 39.25, 114.42, 114.88, 115.24, 123.05, 126.17, 127.64, 127.68, 129.08, 130.39, 130.47, 134.77, 137.04, 137.58, 137.91, 146.37, 154.44, 171.32. Anal. Calcd for C_22_H_24_O_3_ C, 78.54; H, 7.19; Found: C 78.56, H 7.17.

**Compound 12**: ^1^H NMR (CDCl_3_) δ 1.09 (t, *J* = 7.2 Hz, 3H);1.86 (m, 2H); 2.60 (t, *J* = 7.2 Hz, 2H); 3.37 (m, 4H); 5.06–5.14 (series of m, 4H); 5.96 (m, 2H); 6.87 (d, *J* = 8.4 Hz, 1H); 7.05 (m, 2H); 7.15 (d, *J* = 8.4 Hz, 1H); 7.37 (m, 2H). ^13^C NMR (CDCl_3_) δ 13.78, 18.51, 34.70, 36.18, 39.40, 115.63, 116.01, 116.60, 127.43, 128.25, 129.12, 130.40, 131.18, 132.15, 132.60, 135.45, 135.62, 137.77, 148.42, 151.03, 172.28. Anal. Calcd for C_22_H_24_O_3_ C, 78.54; H, 7.19; Found: C 78.52, H 7.20.

### Cell culture

#### HFF1 cell culture

HFF1 are foreskin fibroblast cells type 1, purchased from ATCC (USA). Cells were cultured and expanded in high glucose Dulbecco’s modified Eagle’s medium (DMEM) supplemented with 10% foetal bovine serum (FBS), 400mM glutamine, 100 U/ml penicillin and 100 μg/mL streptomycin at 37°C in a humidified atmosphere containing 5% CO_2_. Only cells from passages 2–3 were used. The cell culture medium was refreshed every 3 days.

#### HepG2 cell culture

HepG2 is a hepatic cancer cell line, purchased from ATCC (USA). Cells were cultured and expanded in low glucose Dulbecco’s modified Eagle’s medium (DMEM) supplemented with 10% foetal bovine serum (FB, Gibco Life technology), 100 U/mL penicillin and 100 μg/ml streptomycin at 37°C in a humidified atmosphere containing 5% CO_2_. Exclusively cells from passage 3 were used. The cell culture medium was refreshed every 3 days.

#### Compounds preparation for bioassay

A pre-working solution containing 10 mM of magnolol **1** and honokiol **2** derivates was dissolved in dimethyl sulfoxide (DMSO). The working solution was diluted in cultured medium at the concentration of 1 mM and then, directly tested on cultured cells at the linear final concentration of 1, 20, 40, 60 and 80 μM. DMSO was diluted at the same linear final concentrations, in the culture medium and used as control.

#### Cell viability

Cell proliferation was determined using the EZ4U kit, (BIOMEDICA immunoassay) which is based on the 3-(4,5-dimethylthiazol-2-yl)-2,5-diphenyltetrazolium bromide (MTT) assay. Approximately 10 000 HepG2 and HFF1 cells were plated in 96-well plates. Both cell lines have a high proliferation rate. After cell adhesion, cells were starved for 24 h in medium containing 0.5% FBS in order to synchronise them in the G_0_ phase of the cell cycle and test the compounds action.

Synchronised cells medium was changed to normal medium (10% FBS) and cells were treated for 24–48 h with magnolol **1**, honokiol **2** and derivates at the different linear concentrations (1, 20, 40, 60 and 80 μM). At different time points, following magnolol **1**, honokiol **2** and derivates treatment, the medium was removed and 100 μL of EZ4U (20 mL of 5mg/mL solution) were added to each well and incubated at 37°C for 2h. The plates were spun and the purple-coloured precipitates of formazan were detected at 450 nm and 620 nm by TECAN plate reader. Changes in the viability of honokiol and magnolol derivates-treated HepG2 and HFF1 cells were expressed as fold change compared to treated control cells. Control cells were considered to be 100% viable. In order to measure possible interference with DMSO compound solvents, the same concentrations of DMSO without the compound were tested.

Results of the experiments on magnolol **1** and honokiol **2** are shown as mean values plus standard deviations of three independent experiments for 12 technical replicates on two cell lines. All experiments on magnolol and honokiol derivatives **3**–**12** are shown as mean values plus standard deviations of four independent experiments for 12 technical replicates on HepG2 and HFF1.

### Statistical analyses

Statistical analysis was performed using GraphPad Prism software. The experiments were performed four times for each cell line for magnolol and honokiol derivatives **3**–**12** and three times for magnolol **1** and honokiol **2**. In each experiment 12 technical replicates for each compound were performed. Non-parametric one-way ANOVA and student t-test were applied. P < 0.05 was considered statistically significant. Non-parametric one-way ANOVA was used to evaluate the distributions and homogeneity of each group variance for the different compounds. Student t-test was used to evaluate, within the same group, differences among the different concentrations compared to negative controls and DMSO controls.

## Results

### Syntheses of magnolol 1 and honokiol 2 derivatives

**Fig 1:**
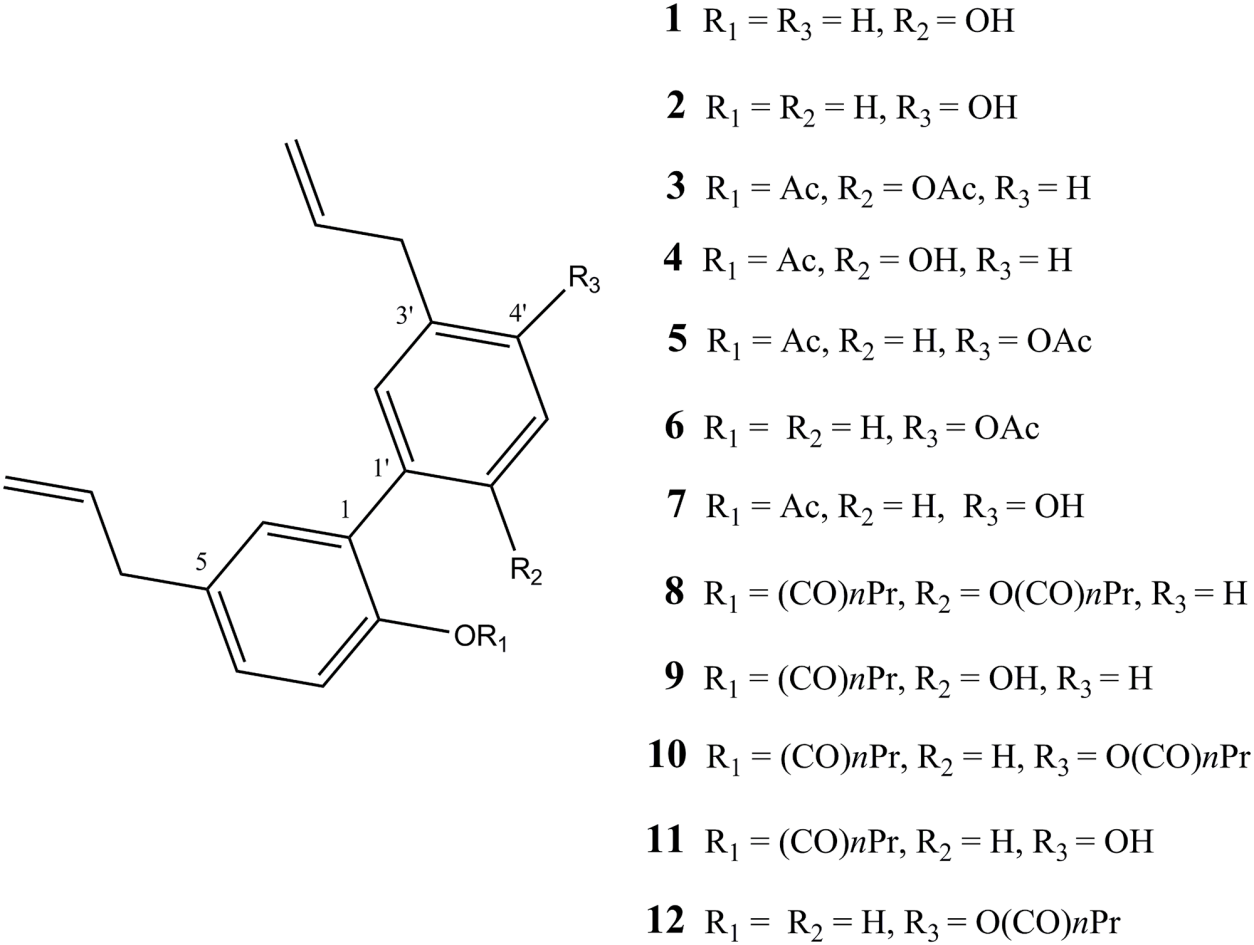
List of the studied compounds.

To investigate the influence of the phenol-OH group of magnolol **1** and honokiol **2** on the permeabilisation of the cell membrane and inhibition of the proliferation of tumoural hepatocytes, compounds **3**–**12** (Fig 1) were prepared.

**Fig 2:**
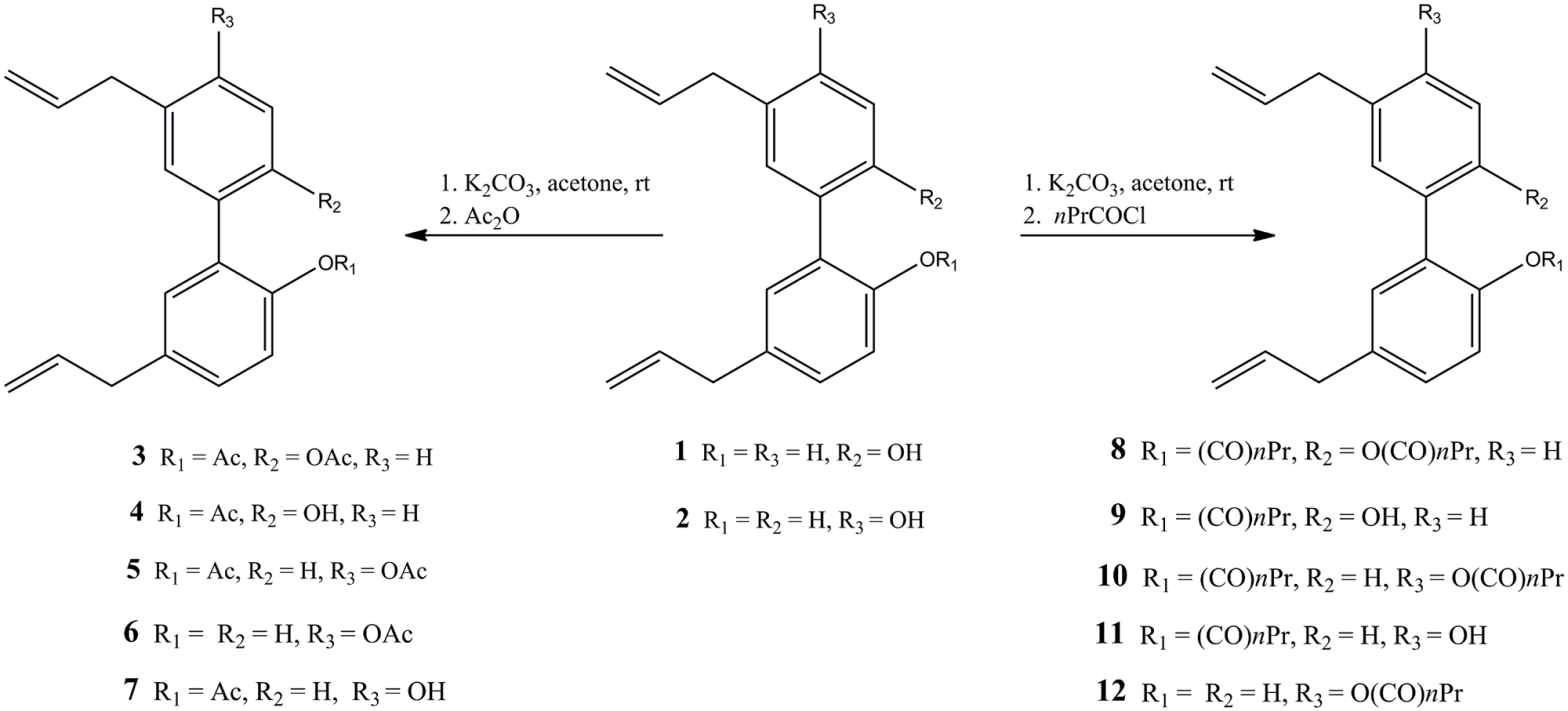
Esterification reactions.

A set of magnolol **1** and honokiol **2** derivatives were synthetised by transformation of the free phenolic OH-groups in ester and diester derivatives, respectively. Diacetate **3** and **5** were obtained in large amount by reaction, at room temperature, of the phenol-OH groups of magnolol **1** and honokiol **2** with two equivalents of potassium carbonate in dry acetone and acetic anhydride, respectively (Fig 2).

Monoacetate **4** was obtained under the same reaction conditions using one equivalent of inorganic base and acetic anhydride. Honokiol monoacetates **6** and **7** were synthetised in satisfactory yield starting from one equivalent of potassium carbonate in dry acetone with the addition, after 30 min, from the start of the reaction, of acetic anhydride. Under these conditions, di- and monoesters **5**, **6** and **7** were obtained in molar ratio 1.3: 3.3: 1, respectively. Although compounds **3**–**5** are known in literature [50, 22], a sustainable inorganic base was used instead of an organic base as generally done for this kind of reaction, allowing for the achievement of higher yields of esters. Di-butyrate derivatives **8** and **10** were obtained by reaction of magnolol **1** and honokiol **2** with two equivalents of dry potassium carbonate in acetone and butyryl chloride. Monobutyrate **9**, **11** and **12** were achieved starting from the corresponding hydroxylated biphenyl by treatment with one equivalent of potassium carbonate and then butyryl chloride. Under these conditions, diester **10** and monoesters **11** and **12** were achieved in molar ratio 1.6: 1: 2.3, respectively. Proton NMR spectroscopy analysis of monoesters **6**, **7**, **11** and **12** compared with ^1^H NMR spectra of known honokiol mono derivatives, allowed for the characterisation of all isomers monosubstituted at phenol-OH group. The nuclear Overhauser effect (nOe) of bare phenol-OH and *ortho* proton of compounds **11** and **12** confirmed both substitution at phenol-OH in position 2 and at phenol-OH in position 4ʹ, respectively. Improved selectivity of monoacyl esters **6** and **7** with diacyl ester **5** and reduced reaction time were obtained under microwave conditions (Table 1) by selecting carbonate as base in acetone and acetic anhydride. Nevertheless, in all experiments the conversion raised only of 20% (determined by ^1^ H NMR) even after longer reaction time.

The reaction was investigated using three different carbonates that reacted with the phenol-OH group forming the alkoxide ion and alkaline bicarbonate. Cesium carbonate was selected due to its high solubility in organic solvent and its high basicity being the most basic of the alkali carbonate of the group I metals. Calcium carbonate would be more effective with sterically demanding substrates having a smaller atomic radius compared to potassium and cesium.

The carbonate anion is a weak base and reacts very slowly with the phenol-OH group allowing for the shifting of the selectivity towards monosubstituted esters. The highest selectivity between mono and diesters was achieved using calcium carbonate and acetic anhydride without solvent.

### Biological evaluation

**Fig 3:**
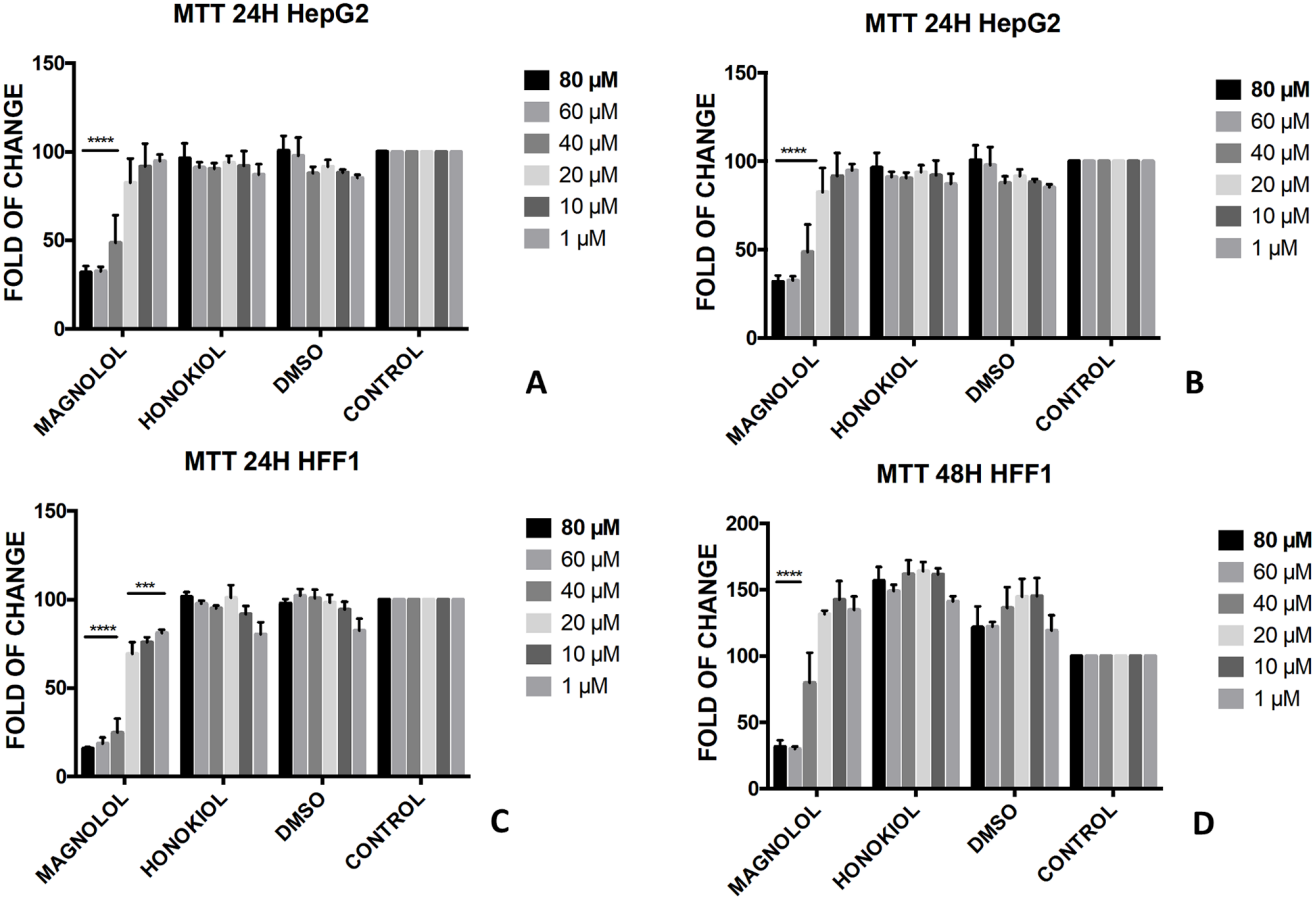
Bioassay of magnolol 1 and honokiol 2. Effect of magnolol **1** and honokiol **2** on HepG2 cells (A and B) and HFF1 cells (C and D) at concentrations of 1–80 μM after 24 and 48 h, respectively. *** = <0.001, **** = <0.0001 *vs*. control.

Magnolol **1** showed the same toxic effect against both HFF1 and HepG2 at concentration below 40 μM even after 24h (Fig 3A and 3B). On the contrary, no effect was observed with honokiol **2** in both cell lines after 24h and 48h treatment at concentrations between 1–80 μM (Fig 3C and 3D).

**Fig 4:**
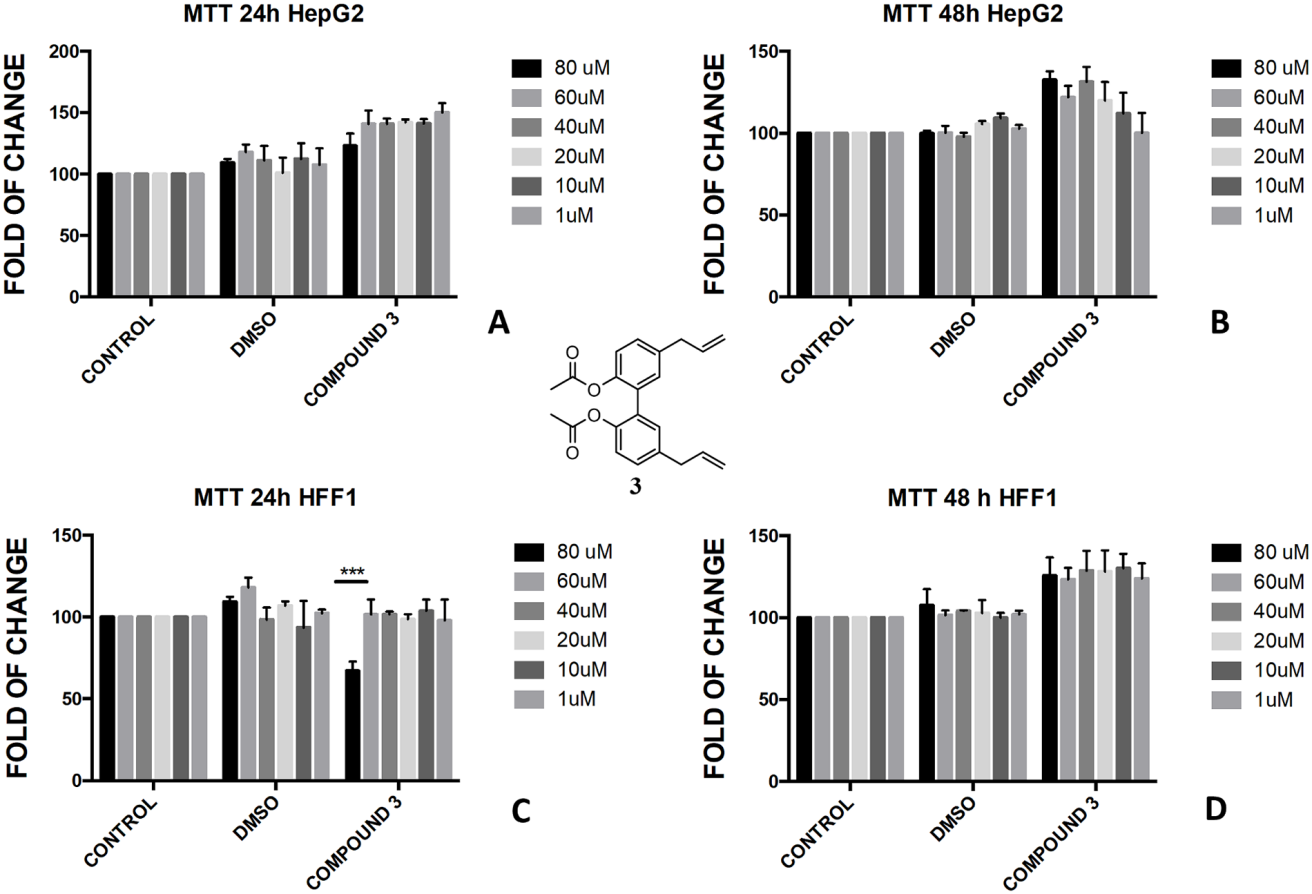
Bioassay of compound 3. Effect of magnolol diacetate **3** on HepG2 cells (A and B) and HFF1 cells (C and D) at concentrations of 1–80 μM after 24 and 48 h, respectively. *** = <0.001 *vs*. control.

**Fig 5:**
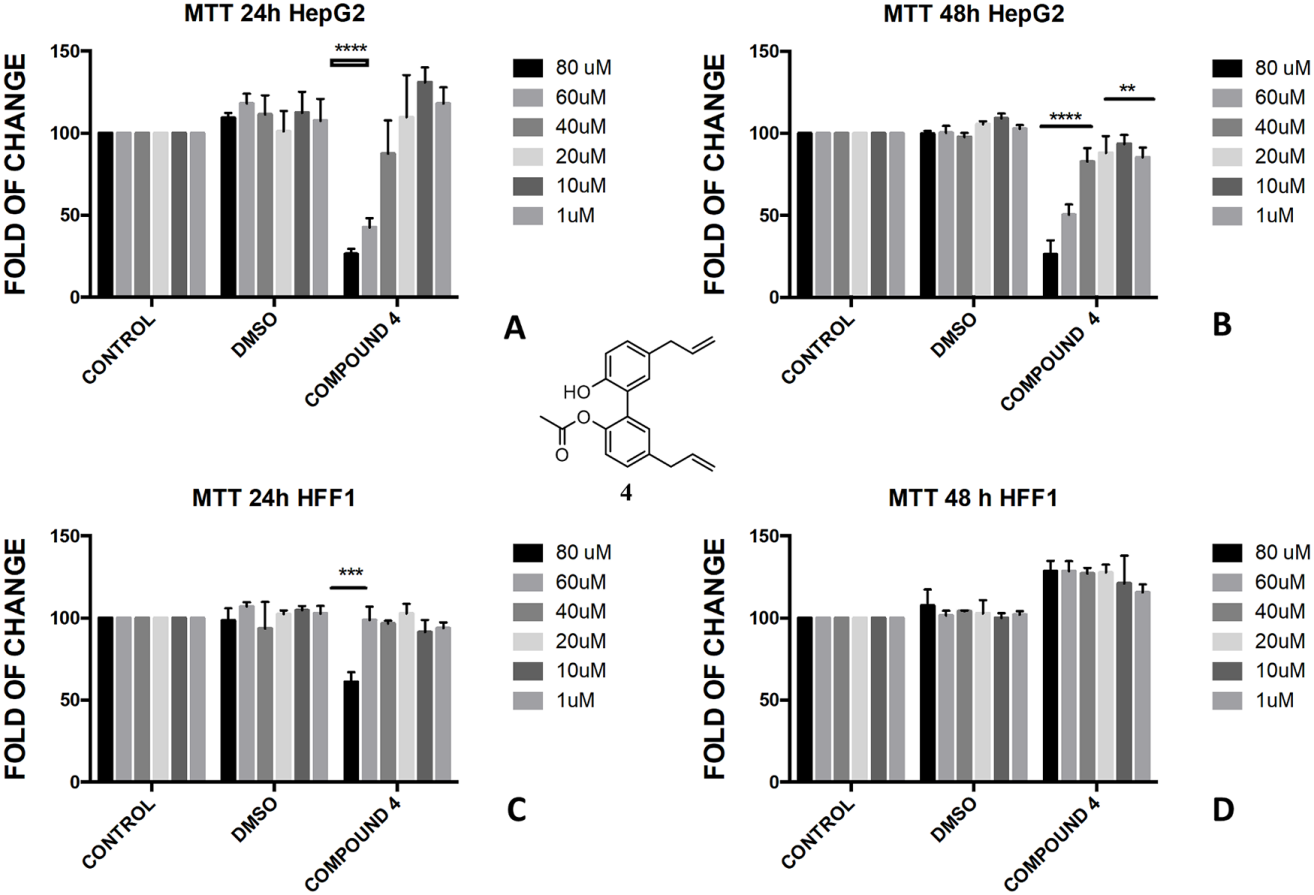
Bioassay of compound 4. Effect of magnolol monoacetate **4** on HepG2 cells (A and B) and HFF1 cells (C and D) at concentrations 1–80 μM after 24 and 48 h, respectively. ** = <0.01,*** = <0.001, **** = <0.0001 *vs*. control.

**Fig 6:**
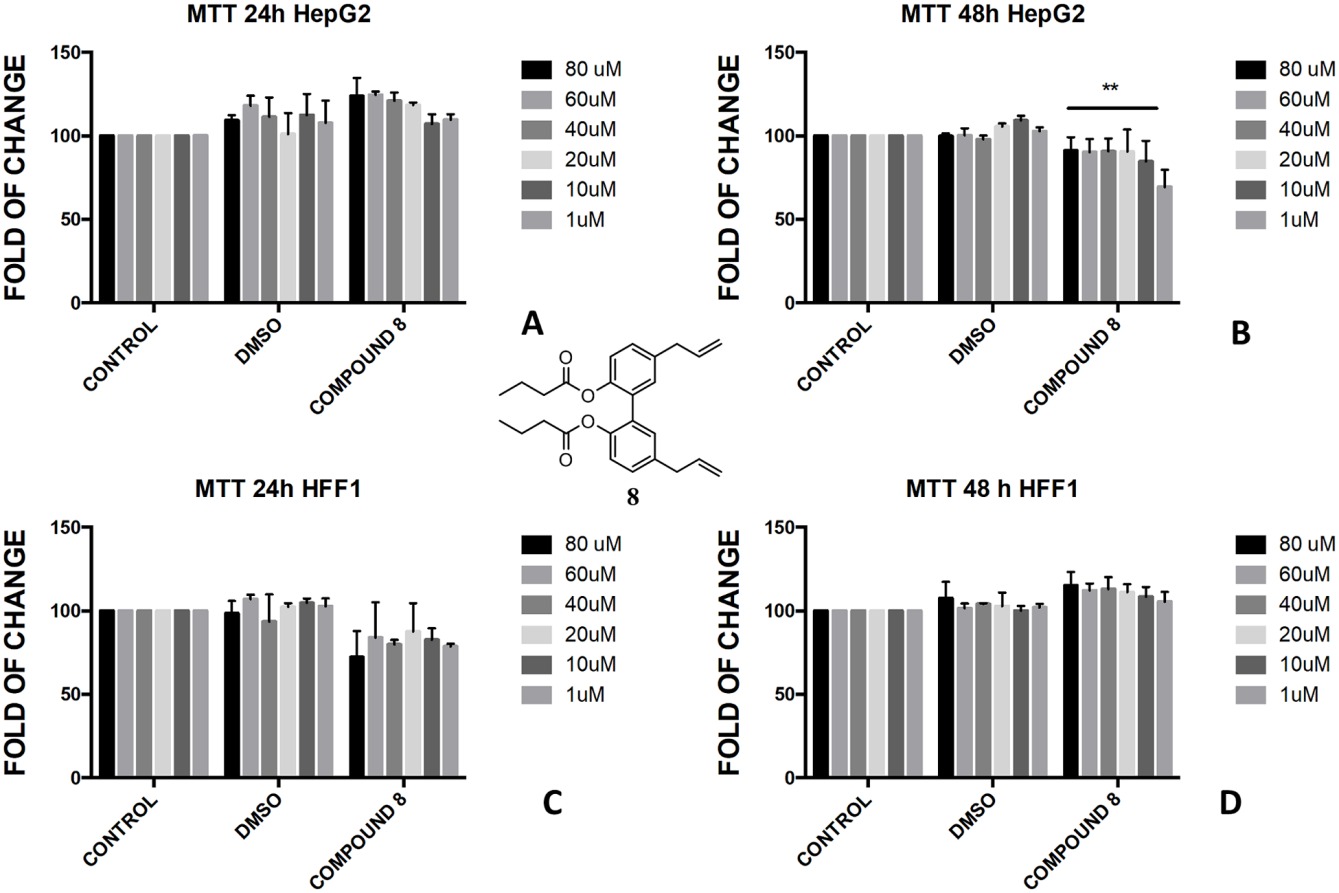
Bioassay of compound 8. Effect of magnolol diacetate **8** on HepG2 cells (A and B) and HFF1 cells (C and D) at concentrations of 1–80 μM after 24 and 48 h, respectively. ** = <0.01 *vs* control.

**Fig 7:**
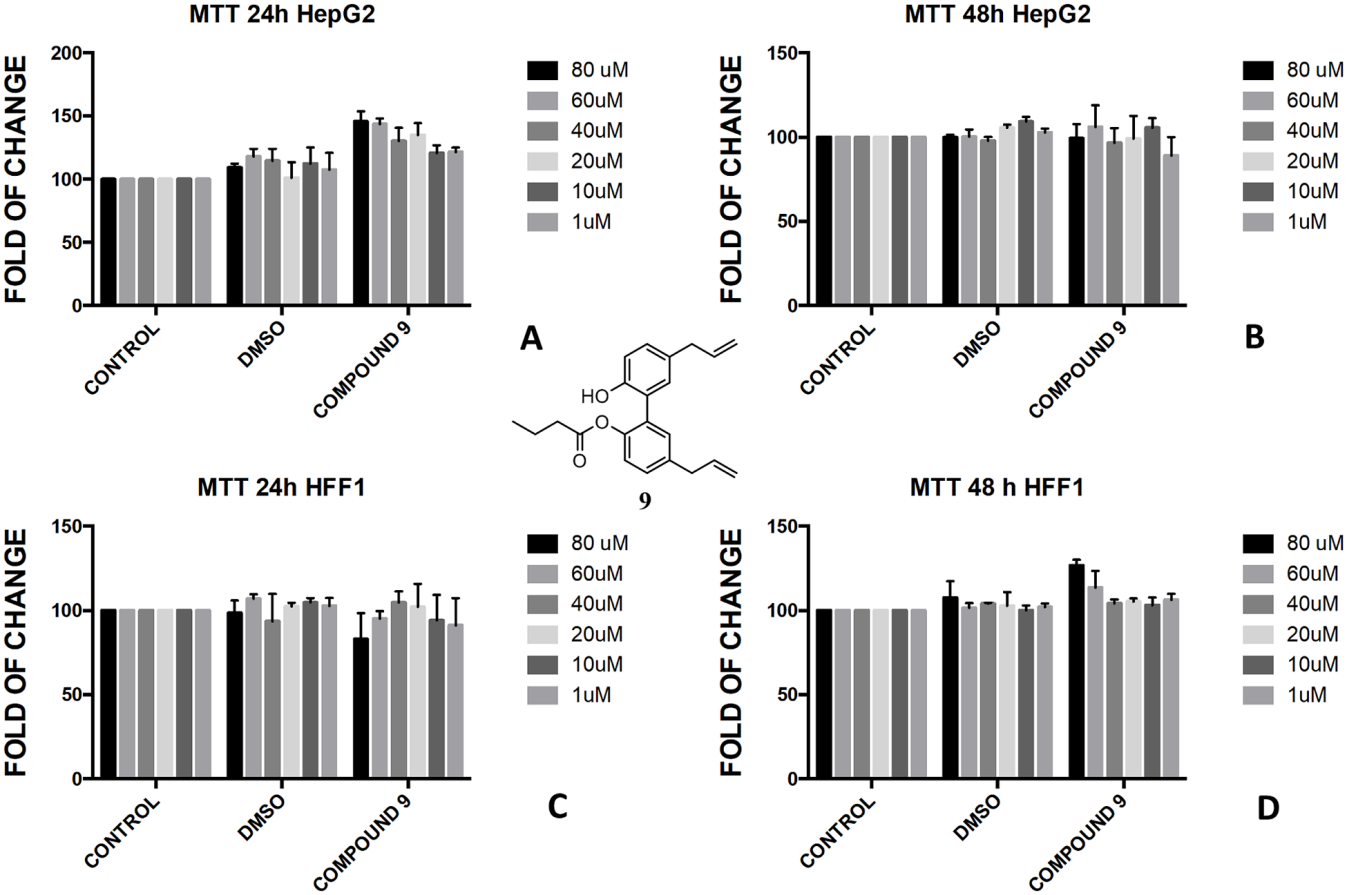
Bioassay of compound 9. Effect of magnolol monobutyrate **9** on HepG2 cells (A and B) and HFF1 cells (C and D) at concentrations of 1–80 μM after 24 and 48 h, respectively.

The protection of phenol-OH groups with an acetyl and a butyrate was the reason for the difference in cell viability and cytotoxicity. Interestingly, when magnolol **1** was mono and diprotected at phenol-OH group with an acetate (compounds **3** and **4**, respectively) or with a butyrate (compounds **8** and **9**, respectively), 100% viability was assessed at 48 h for all tested concentrations showing a complete protective effect of the ester group in non-tumoural cells (HFF1) (Figs 4C, 4D, 5C, 5D, 6C, 6D, 7C and 7D).

**Fig 8:**
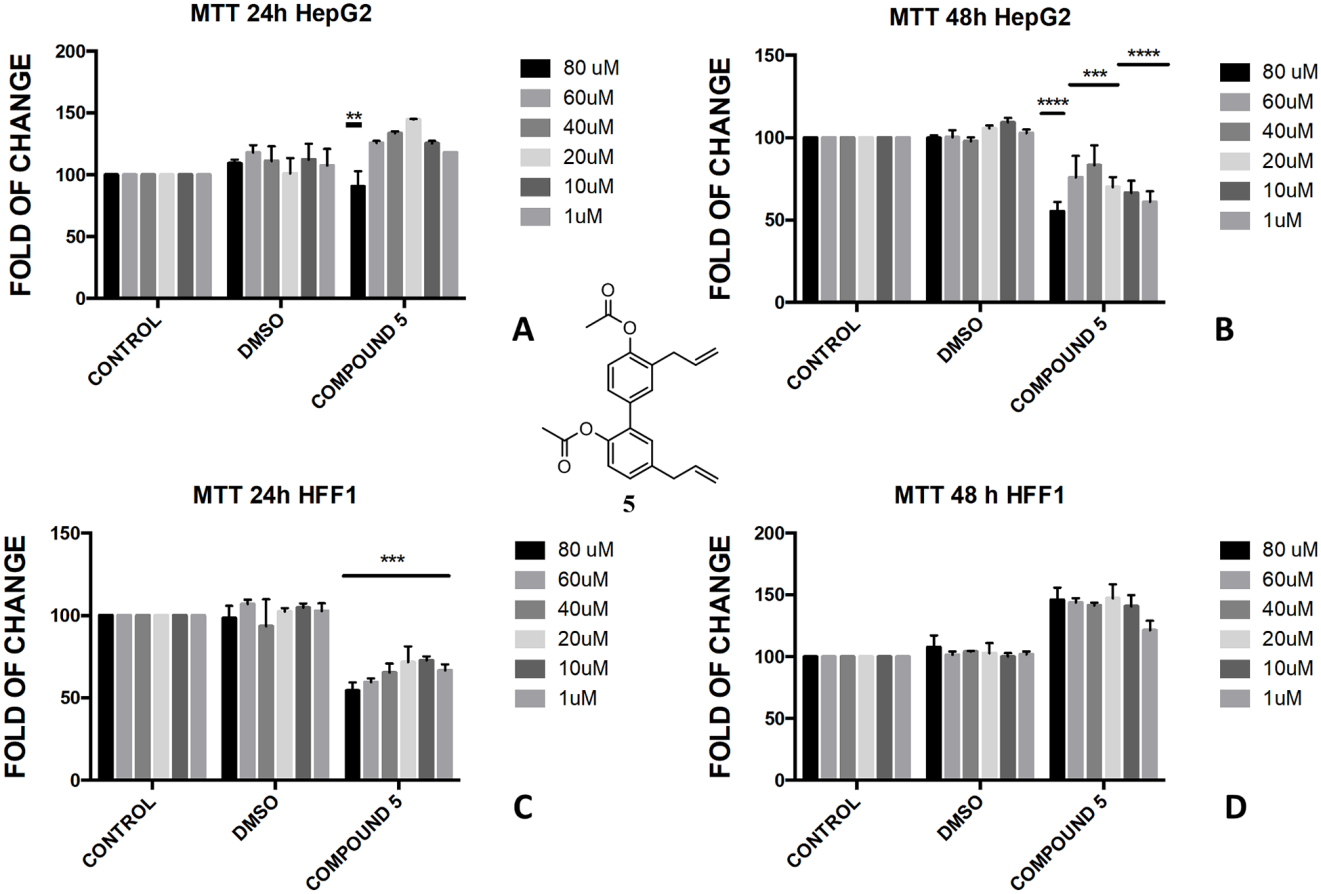
Bioassay of compound 5. Effect of honokiol diacetate **5** on HepG2 cells (A and B) and HFF1 cells (C and D) at concentrations 1–80 μM after 24 and 48 h, respectively. ** = <0.01,*** = <0.001, **** = <0.0001 *vs*. control.

**Fig 9:**
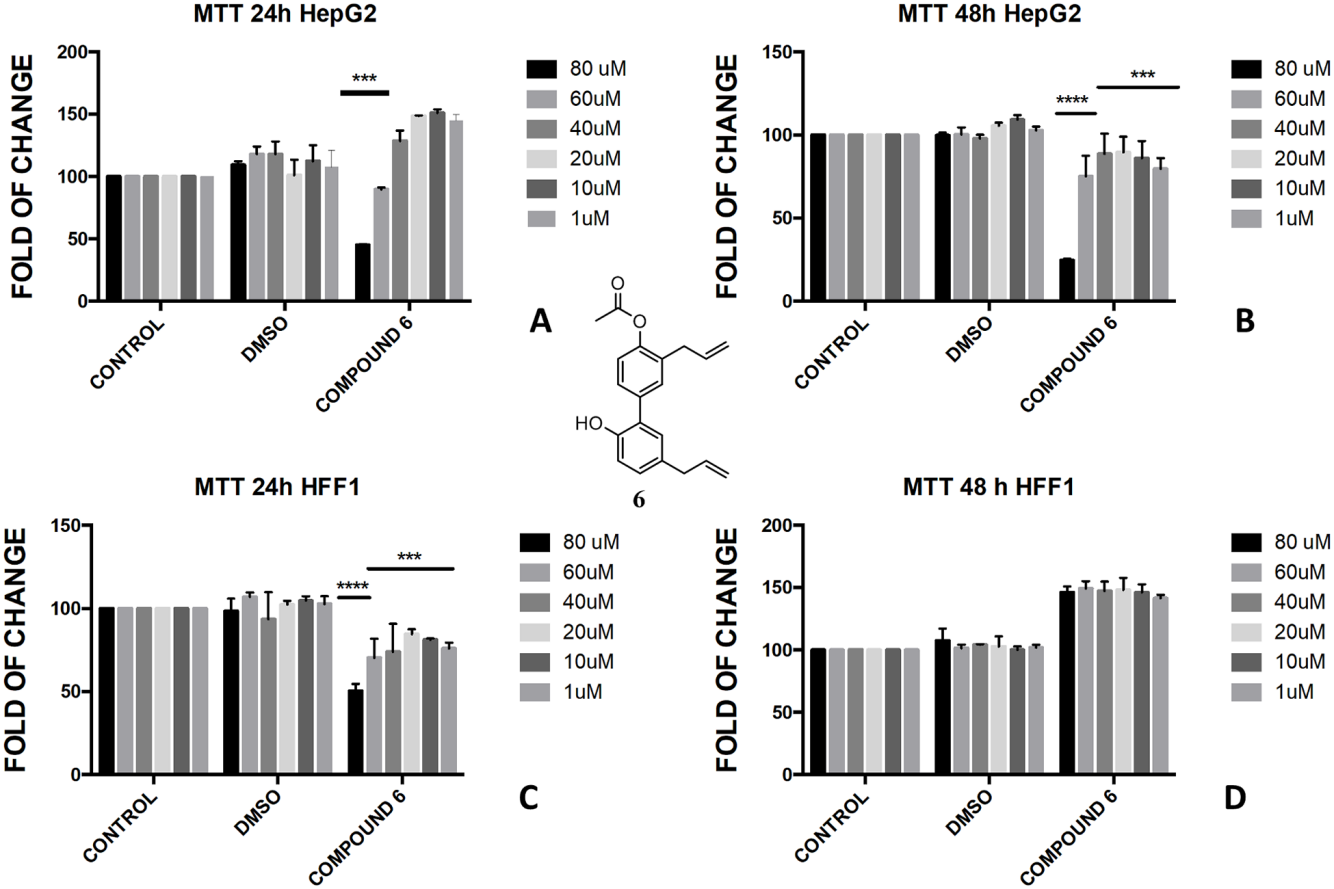
Bioassay of compound 6. Effect of honokiol monoacetate **6** on HepG2 cells (A and B) and HFF1 cells (C and D) at concentrations 1–80 μM after 24 and 48 h, respectively. *** = <0.001, **** = <0.0001 *vs*. control.

The presence of a bare phenol-OH group in compound **4** made the compound toxic in HepG2 cells at concentration above 40 μM (Fig 5A and 5B), whereas no effect was observed in di-substituted magnolol acetate **3** (Fig 4A and 4B). On the contrary, toxic effect was assessed when honokiol diacetate **5** (Fig 8A and 8B) and monoacetate **6** (Fig 9A and 9B) were added to HepG2 cells as shown by the higher cytotoxicity of the 48h treatment with the diester **5**.

**Fig 10:**
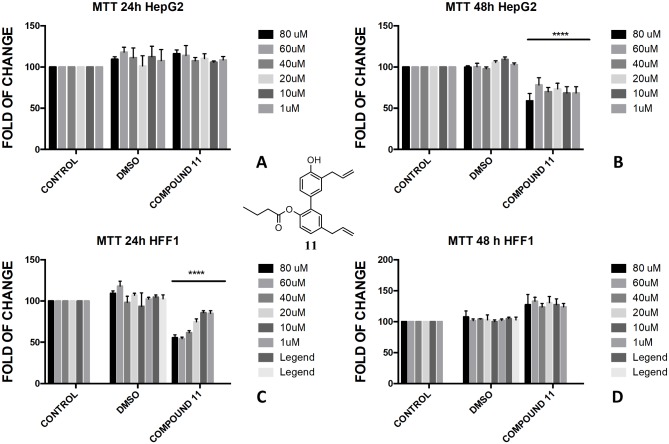
Bioassay of compound 11. Effect of honokiol monobutyrate **11** on HepG2 cells (A and B) and HFF1 cells (C and D) at concentrations of 1–80 μM after 24 and 48 h, respectively. **** = <0.0001 respectively *vs*. control.

**Fig 11:**
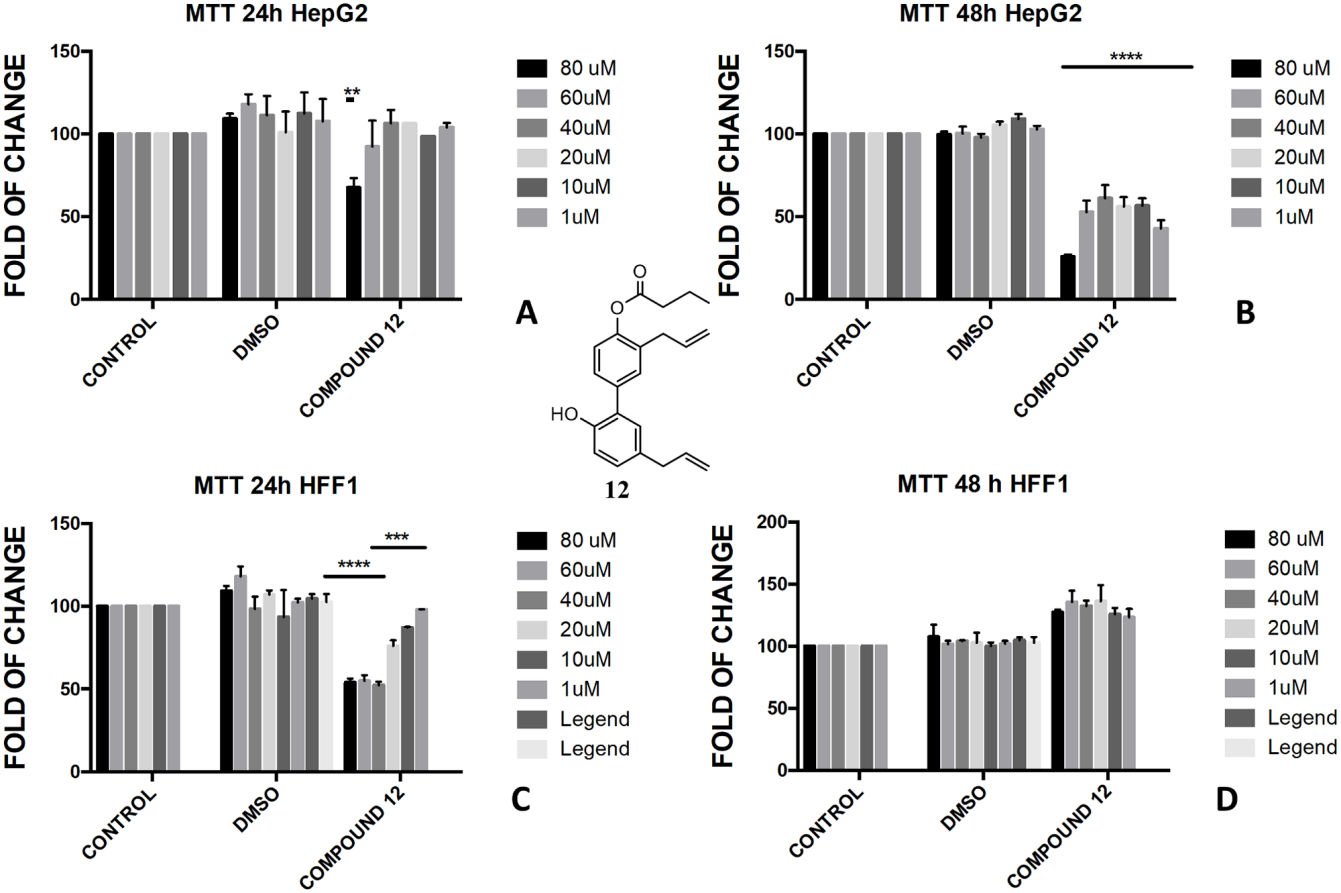
Bioassay of compound 12. Effect of honokiol monobutyrate **12** on HepG2 cells (A and B) and HFF1 cells (C and D) at concentrations of 1–80 μM after 24 and 48 h, respectively. ** = <0.01,*** = <0.001, **** = <0.0001 *vs*. control.

**Fig 12:**
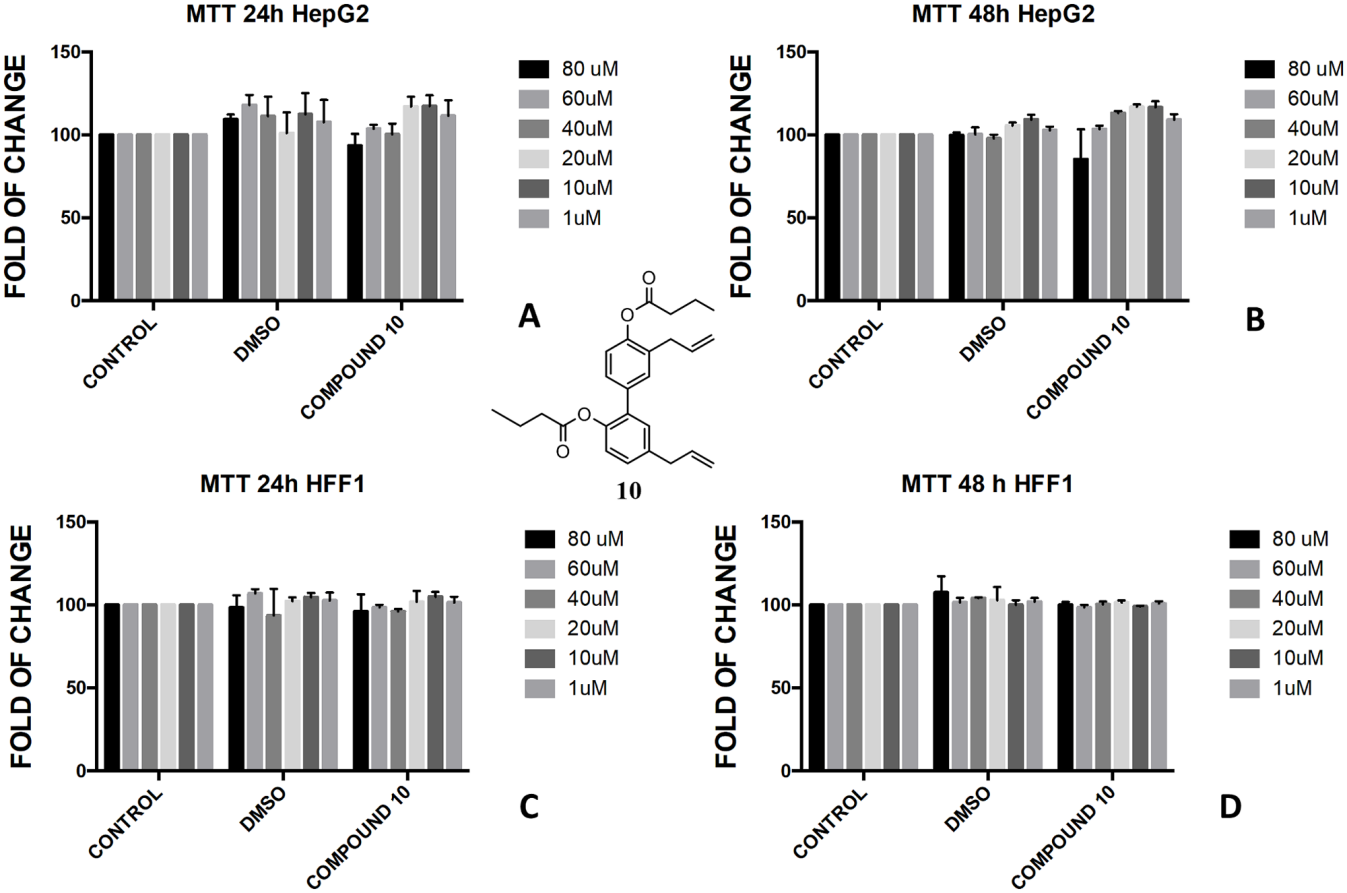
Bioassay of compound 10. Effect of honokiol dibutyrate **10** on HepG2 cells (A and B) and HFF1 cells (C and D) at concentrations of 1–80 μM after 24 and 48 h, respectively.

In the butyrate series, the effect of magnolol and honokiol derivatives changed remarkably. Magnolol mono- and dibutyrate (compounds **9** and **8**, respectively) were not cytotoxic in HepG2 cells (Figs 6A, 6B, 7A and 7B), even though a minor effect was observed after 48h with 1 μM of diester **8** (Fig 6B). Honokiol mono-butyrate **11** and **12** resulted cytototoxic in HepG2 cells at all tested concentrations (Figs 10A, 10B, 11A and 11B). Mono-butyrate **12** was the most cytotoxic, causing the death of 50% of the cell at the concentration of 1 μM. On the contrary, no cytotoxic effect was assessed for diester **10** (Fig 12A and 12B). After 48 h, 100% cell viability was assessed for HFF1 treated with compounds **10**–**12** at all tested concentrations showing a complete protective effect of the ester group in non-tumoural cells (Figs 10D, 11D and 12D).

## Discussion

Recent evidences suggest an important role of antioxidants and anti-inflammatory drugs in fighting liver diseases [51]. In different situation, antioxidants may exert either beneficial or detrimental effects on reactive oxygen species (ROS) homeostasis [52]. Within this context magnolol **1** and honokiol **2**, the bioactive phytochemicals contained in *Magnolia officinalis*, are uncommon antioxidants bearing isomeric bisphenol cores. Antioxidant properties of both biphenyls have been recently studied, highlighting the role of intramolecular and intermolecular interactions and excluding the generation of superoxide radical by reaction with molecular oxygen [18, 53]. Involvement of allyl groups in the antioxidant activity of magnolol **1** and honokiol **2** has been also excluded [53]. Nevertheless, the presence of hydroxylated substituents in magnolol derivatives can affect the antioxidant activity of the resulting compound [18]. Recently, magnolol **1** and honokiol **2** have been used in combination with chemotherapies, improving the activity of the drug [32, 35]. For these reasons, the structure of magnolol **1** and honokiol **2** at the phenolic OH-group was manipulated by ester derivatives using an acetyl or a butyryl group. Ten derivatives of magnolol **1** and honokiol **2** were synthesized (Fig 1) and compared with their parental natural compounds for the ability of inhibiting the proliferation of tumoural hepatic cells (HepG2) and human foreskin fibroblasts (HFF1). Although magnolol **1** and honokiol **2** are regioisomers, the different position of one phenol-OH group conferred them distinct conformations and, thus, diverse reactivity. Phenol-OH groups in magnolol **1** have different acidity due to the formation of an intramolecular H-bond between the two phenol-OH groups that stabilises the mono anion formed in presence of one equivalent of base [53]. Thus, a delayed deprotonation of the second phenol-OH, in the presence of one equivalent of base, allowed us to better control the formation of monosubstituted esters, obtainable in high yield. Moreover, a C_2_-symmetry axis in magnolol **1** allowed for only one monoester isomer. On the contrary, in honokiol **2**, the large dihedral angle and the distance of the two phenol-OH groups reduced the conjugation effect. Moreover similar reactivity towards a base was observed, although, a different acidity was highlighted in a range of pH between 8.6 and 9.8, where the deprotonation is mainly due to the phenol-OH in 2-position [54]. At higher pH, the two phenol-OH group in honokiol **2** were both deprotonated, thus, under strong basic conditions, steric effects might influence the reactivity of phenol-OH group favouring the 4ʹ position, as observed in monoprotection of honokiol **2** with acylic group (*e*.*g*. compounds **6** and **12**).

These features reflected also the different activity that monoesters and diesters of magnolol **1** and honokiol **2** exerted in a biological *in vitro* system. Generally, the presence of a free phenol-OH group seemed to be a key point in the cytotoxicity observed in HepG2, as observed for the compounds **4**, **6**, **11** and **12** (Figs 5D, 9D, 10D and 11D). Moreover, a change in polarity of magnolol **1** and honokiol **2**, due to the protection of one phenol-OH group exerted a remarkable effect on their pharmacological activity. Above 40 μM, different behaviour was observed between monoacetyl ester **4** and monobutyryl ester **9**. This was likely due to the presence of flexible aliphatic butyryl chain that allowed for the formation of H-bond between phenol-OH group and oxygen of the ester group in compound **9**, hampering the cytotoxic role of the phenol-OH group. On the contrary, in diacetyl derivate **5**, the observed cytotoxicity (Fig 8D) could be related to the nature of the acyl group or the partial hydrolysis of the diester. Unfortunately, it was not possible to confirm this observation because repeated purification methods to obtain monoderivative **7**, failed. In compounds **11** and **12** the cytotoxic activity was greater than in the other monoesters (Figs 10D and 11D) likely enhanced by a combined effect of honokiol structure and butyryl group. In fact, in honokiol **2**, no cytotoxic effect was observed in our experimental conditions in HepG2 cells and complete viability was assessed in the presence of non-tumoural cells (Fig 3A, 3B, 3C and 3D). On the contrary, in magnolol **1**, roughly same cytotoxicity was observed in both cells lines at concentration above 40 μM.

It is generally recognised the role that ROS and mitochondria membrane play during apoptosis, including hepatocarcinoma [51, 52]. The presence of honokiol structure with one free phenol-OH and one butyrate group in compounds **11** and **12** seemed to be tailored to target the hepatocarcinoma. In addition, both compounds were not toxic for non-tumoural cells in the studied concentration range (Figs 10D and 11D). Recently, it has been observed the role of sodium butyrate as enhancer of ROS in hepatocarcinoma [55, 56] and the role of honokiol as activator of mitochondrial ROS by mitochondrial dysfunction and depolarisation of mitochondrial membrane potential [57]. Recently, a different protective and antioxidant effect of sodium butyrate at a lower concentration (0.3 mM) was described in HepG2 [58]. Interestingly, in our study, the butyrate-embedded compound concentration was extremely lower (1–80 μM) compared to those used by other groups, despite able to induce an antiproliferative effect.

Differently from normal cells, cancer cells grow in hypoxic conditions. Consequently, the irreversible mitochondrial permeability transition, due to the honokiol structure, could cause hepatoma cell death. Although honokiol is generally recognised a potent scavenger of superoxide and peroxyl radicals [59], in hepatocarcinoma cells, honokiol **1** might exert a double role depending on the cellular demand for ROS at a particular stage of cell development. Within this context, it has been previously described a proapoptotic role of honokiol in lung cancer cells by the activation of AMPK and Sirt3-mediated inhibition of hypoxia-inducible factor [60]. A pro-apoptotic role of honokiol **2** was also described in HepG2 cells by a dysregulation of the axis Notch-KRT6B, a pathway strictly related to the malignancy of the hepatocarcinoma [35]. Honokiol was also found to be involved in the inhibition of STAT3 in HCC cells through the inhibition of activation of upstream kinases c-Src, Janus-activated kinase 1 and Janus-activated kinase 2, when used in combination with common chemotherapies [36]. Recently, it has been demonstrated the pro-oxidant action of honokiol in promoting cellular ROS generation in malignant glioma cells and upregulation of SOD expression in *Candida albicans* [61–63]. Therefore, it is not possible to exclude that a suitable combination of both butyric acid and honokiol, in a single structure (*e*.*g*. compounds **11** and **12**) could enhance cytotoxicity in hepatocarcinoma as compared to the parental compounds, leading to a novel pharmacological tool.

Conversely to the literature, according to which the effect of honokiol **1** on HepG2 cells is dose-dependent and time-dependent for concentration higher than 60 μM [35], we found that compounds **11** and **12** exerted a superimposable inhibitory effect on cell proliferation, when used within a range of 1–60 μM. These data suggested that our modified molecules could counteract cells expansion even at very low concentration and only in cancerogenic cells. Thus, we can hypothesize a future use of the described modified compounds for improving pharmacological treatments against liver tumoural diseases in a safety and specific way.

Our results seemed to indicate that the derivatives of honokiol **1** and magnolol **2** could exert a higher anticancer activity, based on their effect on the inhibition of malignant cell proliferation, as compared to the unmodified molecules. Concerning the possible biological signal switched by these molecules, other authors described a role of honokiol **1** in ROS-mediated apoptosis, in SMMC hepatocarcinoma cell line, thus suggesting a possible signalling pathway that involves ROS balancing and mitochondria [57].

## Conclusions

A small set of derivatives of magnolol **1** and honokiol **2**, efficiently prepared under sustainable conditions, in the range between 1–80 μM, affected tumoural hepatocytes cells proliferation while fibrablast cells, used as control for the toxicological effect of the drugs, were unaffected. The pro-drug approach was applied as versatile strategy to improve bioactivity of compounds by careful transformation of the hydroxyl groups of magnolol **1** and honokiol **2** in a suitable ester derivative. The combination of a butyrate ester and a bare phenol-OH group in the honokiol structure played a significant role in the antiproliferative activity and identified an interesting pharmacological lead against hepatocellular carcinoma. Further studies are already being performed in hepato-tumoural cells to identify the molecular effect of these honokiol and magnolol derivatives during cell cycle (G_1_, G_2_ and S check points) and apoptosis.
